# Immobilization of engineered *E. coli* cells for asymmetric reduction of methyl acetoacetate to methyl-(*R*)-3-hydroxybutyrate

**DOI:** 10.1186/s40643-022-00508-4

**Published:** 2022-03-09

**Authors:** Qing-Sheng Chen, Xin Yuan, Fei Peng, Wen-Yong Lou

**Affiliations:** Lab of Applied Biocatalysis, School of Food Science and Technology, South China University of Technology, Guangzhou, 510640 Guangdong China

**Keywords:** Recombinant *E. coli* cell, Immobilization, Asymmetric synthesis, Methyl-(*R*)-3-Hydroxybutyrate

## Abstract

The efficient asymmetric bio-synthesis of chiral *β*-hydroxy esters is of great importance for industrial production. In this work, a simple and productive engineered *E.coli* cell-immobilized strategy was applied for the asymmetric reduction of MAA to (*R*)-HBME with high enantioselectivity. Compared with the corresponding inactivated free cells, the alginate-immobilized cells remained 45% of initial activity at 50 ℃ and 65% after reuse of 10 times. After 60 days of storage at 4 ℃, the immobilized cells maintained more than 80% relative activity. Immobilization contributed significantly to the improvement of thermal stability, pH tolerance, storage stability and operation stability without affecting the yield of product. The immobilized recombinant *E. coli* cell had absolute enantioselectivity for the asymmetric reduction of MAA to (*R*)-HBME with *e*.*e*. > 99.9%. Therefore, microbial cell immobilization is a perspective approach in asymmetric synthesis of chiral *β*-hydroxy esters for industrial applications.

**Graphical Abstract:**

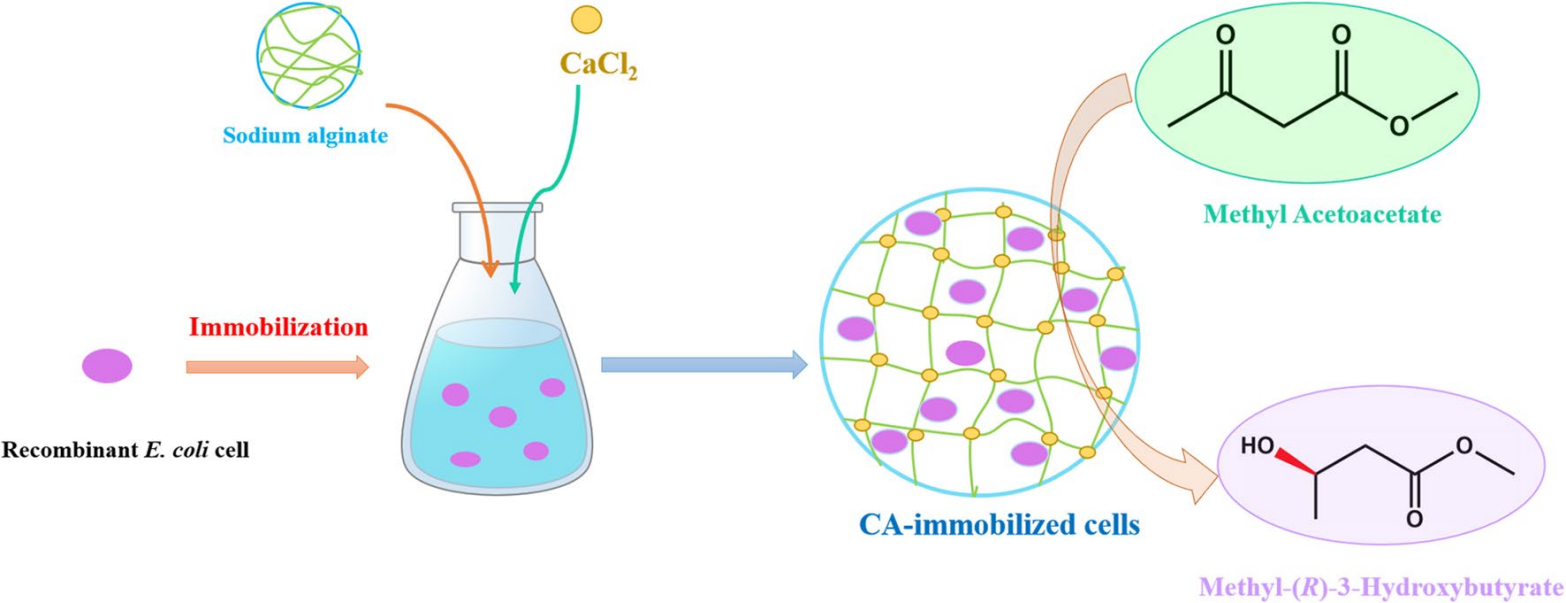

## Introduction

Reduction of prochiral carbonyl compounds to chiral alcohols is a perspective transformation, which is widely applied in industrial application for chiral pharmaceuticals, functional materials, flavors, as well as agricultural chemicals (Luo et al. 2017). For example, methyl-3-hydroxybutyrate (HBME), synthesized from methyl acetoacetate (MAA) with the intramolecular reduction of 3-carbonyl to hydroxy, features the building block for polymers possessing good biocompatibility and controllable biodegradable ability (Thomas et al. 2002; Hollmann et al. 2011). Besides, (*R*)-HBME is a participant in synthesis of *β*-lactam antibiotics, carotenoids and tumor necrosis factor *α*-converting enzyme inhibitors (Guo et al. 2010; Donova et al. 2012; Xie et al. 2007).

Biocatalysis has become the popular method for pharmaceutical intermediates and chemical synthesis with alarming speed due to environmentally friendly, mild reaction conditions and high efficiency. Particularly, unparalleled enzyme specificity is more available than chemical processes to prepare chiral compounds with high purity (Carvalho 2011; Domach 2015). Generally, biocatalysts mainly divide into isolated enzymes and whole cells. Free oxidoreductases are commonly coupled with surprisingly expensive cofactors such as NAD(P)H, whereas whole cells can fulfill cofactors regeneration with sacrificing for low-priced co-substrates to in situ. Some microbes, including bacterium and fungus, possess the catalytic effect of whole cell. A baker’s yeast was reported to be capable of asymmetric reduction of MAA (100 mM) to (*S*)-HBME with moderate enantioselectivities (86.3%) and the target product yield of 32.8% (Xu et al. 2014). *Acetobacter pasteurianus* GIM1.158 was also described as an efficient biocatalyst for asymmetric reduction of MAA (10 mM) to (*R*)-HBME with the high yield (92.9%) and *e.e.* (97.2%) under the optimum reaction condition (Bickerstaff 2006). Through a revolution in genetic technology, chassis cells constructed by cloning multiple target genes were shown to be an ultra-efficient biocatalyst for the synthesis of chiral compounds. The concept is supported by an engineering *E. coli* cell catalyzing MAA (40 mM) reducing to *(R)-*HBME with high enantioselectivities (approximately 100%) (Chen et al. 2012). Moreover, our group successfully overexpressed an AcCR (carbonyl reductase) gene from *Acetobacter* sp. CCTCC M209061 and a GDH (glucose dehydrogenase) gene from *Bacillus subtilis* 168 in an *E. coli* cell. The recombinant *E. coli* cell efficiently reduced high substrate concentration (35 mM) of EAA to (*R*)-EHB (yield of 82.6%) and constantly maintained > 99.9% *e.e.* in any investigated variables (Wang et al. 2013).

However, there are some barriers for industrial reduction using free fragile *E. coli* whole cells as the catalyst, such as poor pH stability, reusability, substrate inhibition and mechanical damage. Immobilization strategy is an ideal approach to compensate for the deficiencies of free cells under drastic reaction conditions. Previous studies also proved biocompatible calcium alginate (Ca-alginate) beads exert positive effects on biotransformations, and the coating shield chitosan in a complex entrapment-encapsulation can further enhance its mechanical properties and anti-swelling properties (Wei et al. 2015; Chen 2013). Therefore, immobilization of cells has a great industrial application prospect for catalytic synthesis. In this work, the immobilized cells were prepared by using the Ca-alginate and Ca-alginate chitosan as support. The catalytic performances of three biocatalysts, including free, Ca-alginate-immobilized and Ca-alginate chitosan-immobilized recombinant *E. coli* cells, were evaluated. Moreover, the reaction conditions were optimized at high concentration for the catalytic ability of asymmetric reduction of MAA as the model substrate.

## Materials and methods

### Strains and reagents

The recombinant *E. coli* cell was prepared and determined its initial reaction rate, yield, enantiomeric purity and relative activity according to our previous methods (Wei et al. 2017)*.* MAA, (*R/S*)-HBME and *tert*-butyldimethyl chlorosilane (TBDMCS) were purchased from Sigma-Aldrich (USA). All other reagents and solvents were of analytical grade and used without further purification.

### Cultivation of *E. coli* BL21(DE3)-pETduet1-*gaccr*-*gdh* cells

*E. coli* BL21(DE3)-pETduet1-*gaccr*-*gdh* strain culture: *E. coli* BL21(DE3)-pETduet1-*gaccr*-*gdh* was inoculated into 50 mL LB broth with 100 μg/mL ampicillin and cultured at 37 ℃ and 160 rpm for 12 h. Secondly, 1 mL of the strain liquid was added to 100 mL of LB broth with 100 μg/mL ampicillin and incubated at 37 ℃ and 160 rpm for approximately 4 h. When the optical density at 600 nm (OD 600) of the culture reached 1.2, the temperature was changed to 20 ℃, and then 40 μL IPTG (500 mM) was injected. Subsequently, the cells were continued to incubate at 20 ℃ and 160 rpm. After18 h of incubation, the cells were harvested by centrifugation (8000 rpm, 4 ℃, 5 min) and washed twice with normal saline.

### Immobilization of *E. coli* BL21(DE3)-pETduet1-*gaccr*-*gdh* cells

120 mg the engineering cells (wet weight) and 4 mL sodium alginate solution (2.5%, w/v) were mixed. Then, the mixture was dropwise added to 2% (w/v) CaCl_2_ solution using a 5 mL syringe. After hardened for 30 min, the beads were collected and washed with Tris–HCl buffer (200 mM, pH 7.0) to obtain calcium alginate-immobilized cells, named as CA-immobilized cells. CA-immobilized cells were added to chitosan solution (0.9%, w/v, pH 5.0) for 20 min, and then were collected by filtration and washed with Tris–HCl buffer (200 mM, pH 7.0) to obtain the immobilized cells with chitosan and calcium alginate, named CS/CA-immobilized cells.

To study the total number of immobilized microbial cells within the beads, three beads were selected at random from a sample (Greenberg et al. 1995). The selected beads were ruptured to make homogenous solution and the solution was diluted 10 times. The cells within the diluted solutions were allowed to grow in petri dishes containing nutrient agar and incubated at 37 ℃ for 24 h. The cell density was obtained by colony on petri dishes.

The catalytic activity of immobilized cells was assessed by the asymmetric reduction of MAA to (*R*)—HBME. The reaction conditions were as follows: 4 mL Tris–HCl buffer (200 mM, pH 7.0), 100 mM MAA, 150 mM glucose, 40 °C, 200 rpm. After the reaction period, 50 μL of sample was taken, and then 200 μL of ethyl acetate was added to extract the substrate and product from the sample. The mixture was shaken with a vortex mixer for 3 min, centrifuged (12,000 r/min, 5 min). Finally, the supernatant was taken and stored at 4 ℃ for GC analysis.

### Preparation of (*R*)-HBME from MAA reduction catalyzed by the immobilized cells

In the typical experiments, the catalysts including the free cells, CA-immobilized cells and CS/CA-immobilized cells were added to 10 mL reactor, respectively. Then, 4 mL Tris–HCl solution (200 mM, pH 7.0) consisting of 100 mM MAA and 200 mM glucose were added above reactor. The reaction was carried out at 40 ℃ and 200 rpm. Aliquots were regularly withdrawn to determine the level of the product during the reaction. The initial reaction rate was calculated according to the level of HBME at 30 min. The yield of product was equal to the determined concentration of (*R*)-HBME divided by the theoretical concentration of (*R*)-HBME.

### Effect of immobilization method on the catalytic performances of the cells.

Based on the above approach preparing (*R*)-HBME, the catalytic performances of the free cells, CA-immobilized cells, and CS/CA-immobilized cells were compared. During the reaction, samples were analyzed to determine the yield of product by gas chromatography.

### Thermal stability of the immobilized cells

The two catalysts including the free cells and CA-immobilized cells were added to 4 mL Tris–HCl solution (200 mM, pH 7.0), respectively, and incubated at the different temperature (30, 40, 50, and 60 ℃) and different time (0, 1, 2, 3, 4, 5, 6 h). The catalytic activity of the catalysts was determined on basis of the above approach in “Immobilization of E. coli BL21(DE3)-pETduet1-gaccr-gdh cells”. The relative activity was calculated as the ratio of the activity of treated CA-immobilized cells exposing to different temperature and pH after required incubation time and activity of the untreated CA-immobilized cells. The activity of the untreated CA-immobilized cells was set as 100%. The relative activity of free cells was calculated in the same way.

### pH stability of the immobilized cells

The pH stability was investigated by pre-incubating the CA-immobilized cells in different pH buffers range of 4.0–8.0 using various buffer systems at 50 mM. The incubated temperature is 4 ℃. The buffers were as follows: citrate–phosphate (pH 4.0–7.0) and Tris–HCl (pH 7.0–8.0). After incubation, immobilized cells were added to reaction mixture solution and reacted for a period of time at 40 ℃. During the incubation process, the catalyst was taken out to determine the catalytic activity according to the above approach.

### Storage stability

The free cells and CA-immobilized cells were stored in Tris–HCl buffer (200 mM, pH 7.0) at 4 ℃. The catalytic activity of the stored catalysts at the different stored time was determined on basis of the above approach.

### Operational stability

The reusability of the free cells and CA-immobilized cells were compared by determining the initial rate during repeated usages. The asymmetric reduction reaction catalyzed by free cells and immobilized cells was 2 h and 4 h per batch, respectively. CA-immobilized cells were obtained by 0.22 μm water microporous membrane filtration, and the free cells were collected by centrifugation (8000 rpm, 5 min, 4 ℃). These deposits were washed twice with Tris–HCl (200 mM, pH 7.0). The recovered catalysts were continued to use for the next batch. The activity of the catalysts in the first cycle was defined as 100%.

### Effect of MAA levels on the biocatalytic synthesis of (*R*)-HBME

4 mL Tris–HCl buffer (200 mM, pH 7.0) reaction systems consisted of CA-immobilized cells containing 240 mg wet cells, MAA, and glucose. The level of MAA ranged from 300 to 700 mM, and the molar ratio of glucose to MAA was at 1:2. The pH of the reaction solution was adjusted with Na_2_CO_3_ and His powder of 2:1 (mass ratio), and the pH was adjusted to 7.0 every 0.5 h. The reaction was conducted at 40 ℃ and 200 rpm. When reaction for 30 min, samples were withdrawn to determine the initial rate, and after the reaction completed, samples were taken out to measure the yield of the product.

### Analytic method

The product was analyzed by an Agilent 6890 N gas chromatograph equipped with a flame ionization detector (FID). The optimal experimental conditions of gas chromatography were as follows: CP-Chiralsil-Dex-CB (USA) column, the inlet temperature at 190 °C, column temperature at 90 °C and detector temperature at 210 °C; flow rate of carrier gas at 1.6 mL/min; split ratio of 30:1. The retention time of MAA and (*R*)-HBME was 4.85 and 6.11 min, respectively.

## Results and discussion

### Effects of immobilization method on the catalytic performances of *E. coli* BL21(DE3)-pETduet1-*gaccr*-*gdh*

Free microbial cells have been reported to exhibit many disadvantages in biocatalytic reactions, including instability, deactivation and poor recyclability (Chen et al. 2015). The immobilized cells could effectively overcome the above shortcomings and meet standards of industrial applications and had its peculiar catalytic performance, but they had a greater impact on the initial reaction rate, yield and the product *e*.*e*. of the biocatalytic reaction (Zhang et al. 2004; Al-Hasawi et al. 2020; Güngörmüşler et al. 2021). Therefore, this study compared the effects on the asymmetric reduction of MAA catalyzed by free recombinant *E. coli* cells and immobilized cells.

**Fig. 1:**
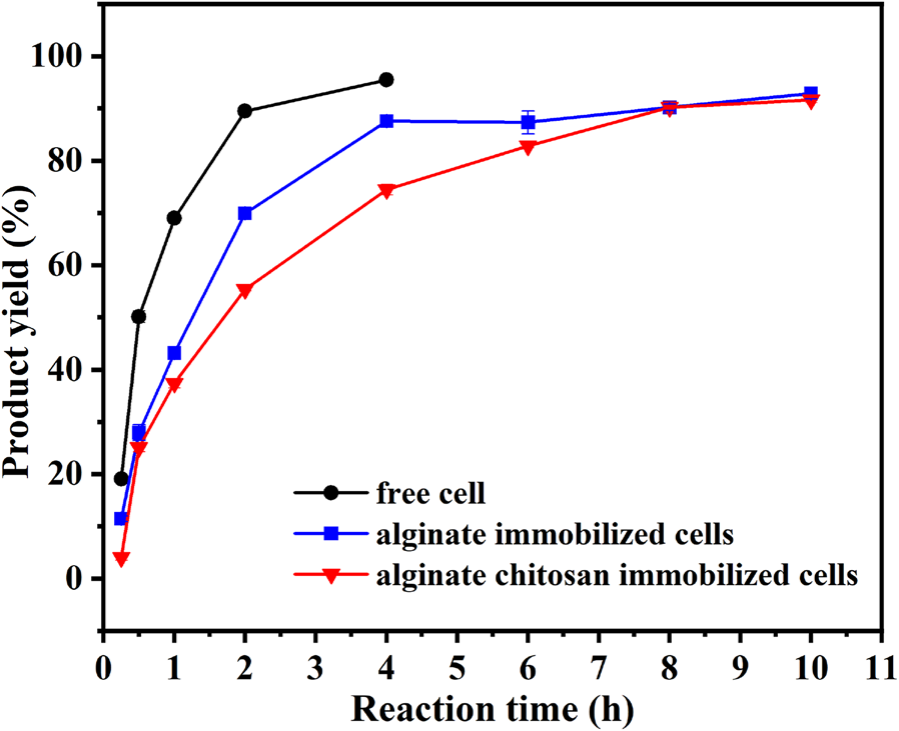
Reaction course curve of the asymmetric reduction of MAA to (*R*)-HBME catalyzed by free, alginate-immobilized and alginate chitosan-immobilized recombinant *E. coli* cells. Reaction conditions: 4 mL Tris–HCl buffer (200 mM, pH 7.0), 100 mM MAA, 150 mM glucose, 30 mg/mL of free recombinant *E. coli* cells (wet weight), or alginate-immobilized cells or the alginate chitosan-immobilized cells containing the same amount of free cells, 40 °C, 200 rpm

As shown in Fig. 1, the initial reaction rate of free recombinant *E. coli* cells catalyzing the asymmetric reduction of MAA to (*R*)-HBME was significantly faster than that of immobilized cells. After 2 h, MAA was reduced almost completely by free *E. coli* cells with the maximum yield of 92%. It took 4 h by the immobilized *E. coli* cells and the maximum yield reached 91%, while 8 h were needed by the alginate chitosan-immobilized cells with the yield of about 90%. A decrease in the reaction rate of the asymmetric reduction may be caused by the increment of mass transfer resistance of substrate absorption and product release when cells entrapped into Ca-alginate beads. Second, the concentration of substrates around the hydrophilic calcium alginate-immobilized microspheres was lower than that of free cells. Furthermore, the reaction rate of alginate chitosan-immobilized cells was the worst among the catalytic reactions of the three biocatalysts. This may be because cells embedded in Ca-alginate beads tightly wrapped with a layer of chitosan to enhance mass transfer resistance both substrate absorption and product release, greatly retarding the reaction rate of asymmetric reduction. The *e*.*e*. of the product still remained > 99.9%, indicating that the recombinant *E. coli* strain had absolute enantioselectivity for the asymmetric reduction of MAA to (*R*)-HBME. Furthermore, a previous study by our group found that the immobilized *Acetobacter* sp. CCTCC M209061 strain was significantly better than free cells in terms of stability and reusability when reducing *β-*ketone esters (Xu et al. 2014). Therefore, it was essential to compare the difference of stability between free and immobilized recombinant *E. coli* cells.

### Effect of temperature on asymmetric reduction of MAA

**Fig. 2:**
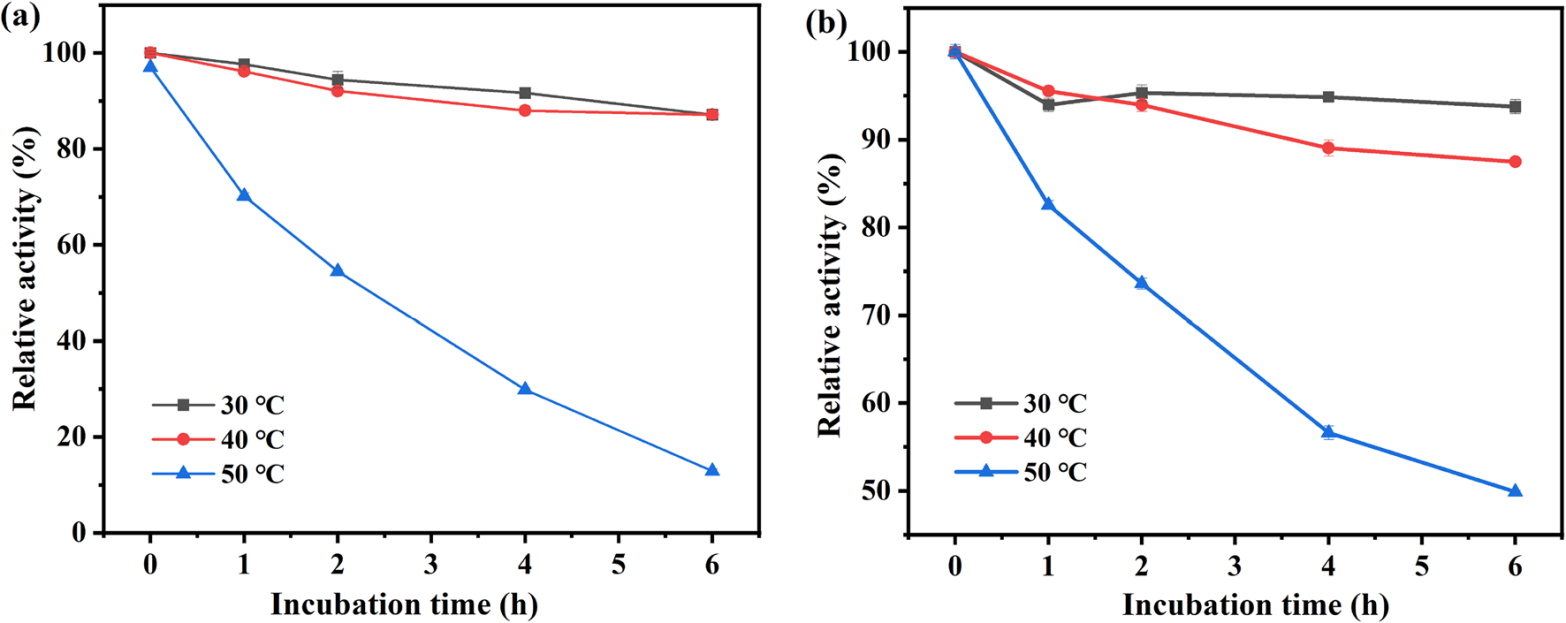
Thermal stability of free (**a**) and alginate-immobilized (**b**) recombinant *E. coli* cells. Reaction conditions: 4 mL Tris–HCl buffer (200 mM, pH 7.0), 100 mM MAA, 150 mM glucose, 30 mg/mL of free recombinant *E. coli* cells (wet weight), or alginate-immobilized cells containing the same amount of free cells, 40 °C, 200 rpm

The recombinant *E. coli* cells embedded with Ca-alginate were incubated at 30, 40, 50, and 60 ℃, respectively. And, the differences between free and immobilized cells were studied on the catalytic ability of MAA to (*R*)-HBME (shown in Fig. 2).

After 6 h of incubation at 30 and 40 °C, both free and immobilized cells could remain more than 90% of initial catalytic activity, respectively. However, incubating at 50 °C, free cells almost completely inactivated but immobilized cells only decreased to 45% of its initial activity. This phenomenon is probably because free cells are incubated at high temperature, causing cell wall separation and death, while immobilized cells are protected by the immobilization carrier. The outer Ca-alginate layer can provide a relatively safe microenvironment for microbial cells, thus weakening the effect of external temperature changes on asymmetric reduction (Huang et al. 2007). On the other hand, the stronger structure of immobilized cells can conduce to enhance tolerance against high temperature (Ou et al. 2012). It can be seen that the thermal stability of immobilized recombinant *E.coli* cells significantly surpassed that of free cells.

### Effect of pH on asymmetric reduction of MAA

**Fig. 3:**
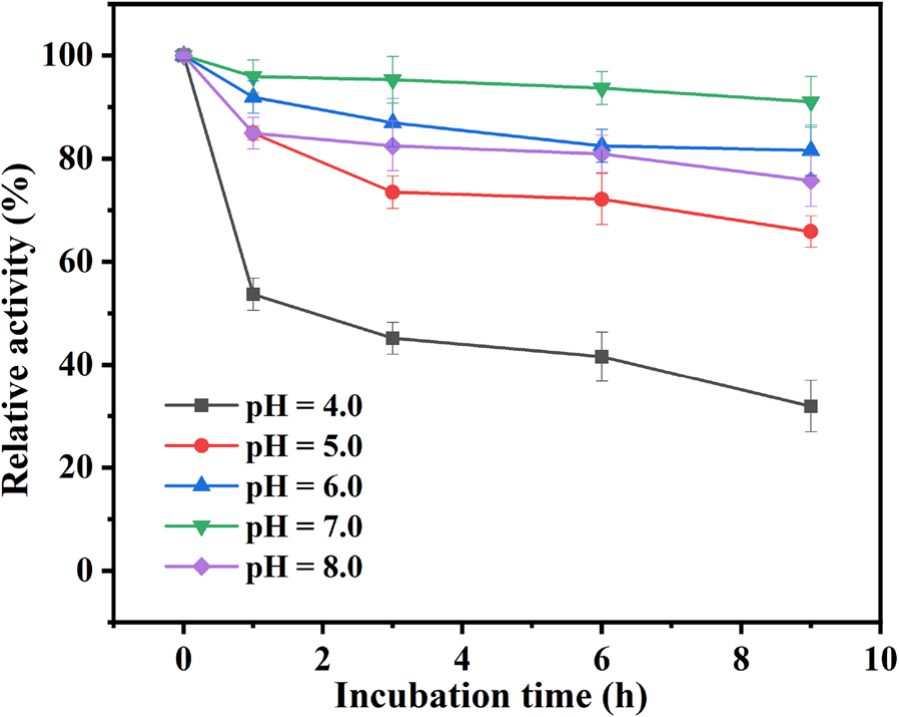
pH stability of immobilized recombinant *E. coli* cells. Reaction conditions: 4 mL Tris–HCl buffer (200 mM, pH 7.0), 100 mM MAA, 150 mM glucose, 30 mg/mL of immobilized recombinant *E. coli* cells (wet weight), 40 ℃, 200 rpm

Since the accumulation of gluconic acid, the by-product, as a kind of a weakly acidic substance would cause a sharp drop in the pH of the reaction system, thus it was fundamental to investigate the stability of recombinant *E. coli* cells incubating in different buffer pH (shown in Fig. 3).

It was shown that the relative activity of immobilized cells had decreased to a certain extent after incubating for 9 h with a range of pH 4.0 to 8.0. The immobilized cells lost approximately 75% of initial activity during 9 h of incubation in pH 4.0, whereas the free cells had totally lost their catalytic activity as aforementioned research (Wei et al. 2017). Therefore, the immobilized cells showed more outstanding pH stability, which may be ascribed to the microenvironmental protection formed by the gel network of immobilized *E. coli* cells from H^+^ poisoning (Erbeldinger et al. 2000).

### Reusability of free and immobilized recombinant *E. coli* cells

**Fig. 4:**
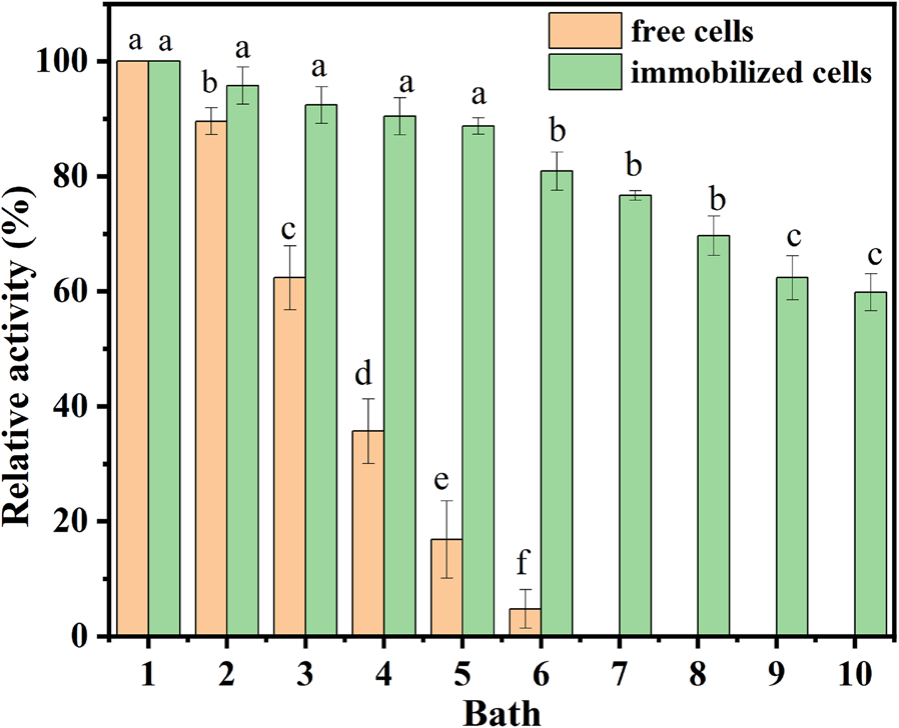
Reusability of free and alginate-immobilized recombinant *E. coli* cells. Reaction conditions: 4 mL Tris–HCl buffer (200 mM, pH 7.0), 100 mM MAA, 150 mM glucose, 30 mg/mL of free recombinant *E. coli* cells (wet weight), or immobilized cells containing the same amount of free cells, 40 ℃, 200 rpm. (*n* = 3, different lowercase letters represent significant differences between groups, *p* < 0.05)

Due to the lower cost, easier reuse of immobilized cells, it was essential to investigate its reusability. Based on the asymmetrically biocatalytic reduction of MAA to (*R*)-HBME, the reusability of free and immobilized recombinant *E. coli* cells was evaluated, as shown in Fig. 4.

The immobilized cells and free cells were separated from reaction mixture by filtration or centrifugation, respectively, and washed twice with Tris–HCl buffer (200 mM, pH 7.0) for the next run. As illustrated in Fig. 4, the reusability of immobilized recombinant *E. coli* cells was much higher than that of free cells in the asymmetric reduction of MAA to (*R*)-HBME. After 6 batches of free cells (each batch for 2 h), the catalytic activity was mostly lost, while the immobilized cells still retained 65% after being reused 10 times (each batch for 4 h). And the particle of immobilized cells was not obviously damaged. Therefore, the operational stability of immobilized recombinant *E. coli* cells was more superlative than that of free cells, and the reused cells had almost no effect on the enantioselectivity of (*R*)-HBME (> 99.9%) yet. The decay of immobilized cells activity due to the following reasons: irreversible structural changes and swelling due to the network partial disintegration; mechanical and electrostatic interactions between cells and matrix, including hydrophobic interaction, intermolecular force, hydrogen bonding force, and others; chemical interactions of matrix with the components of the liquid nutrition medium; the apoptosis cells or denaturation of enzyme during the reaction process and the diffusion limitation by substrate (Levic et al. 2015; Pajic-Lijakovic et al. 2015).

### Storage stability of free and immobilized recombinant *E. coli *cells on asymmetric reduction of MAA to (*R*)-HBME

**Fig. 5:**
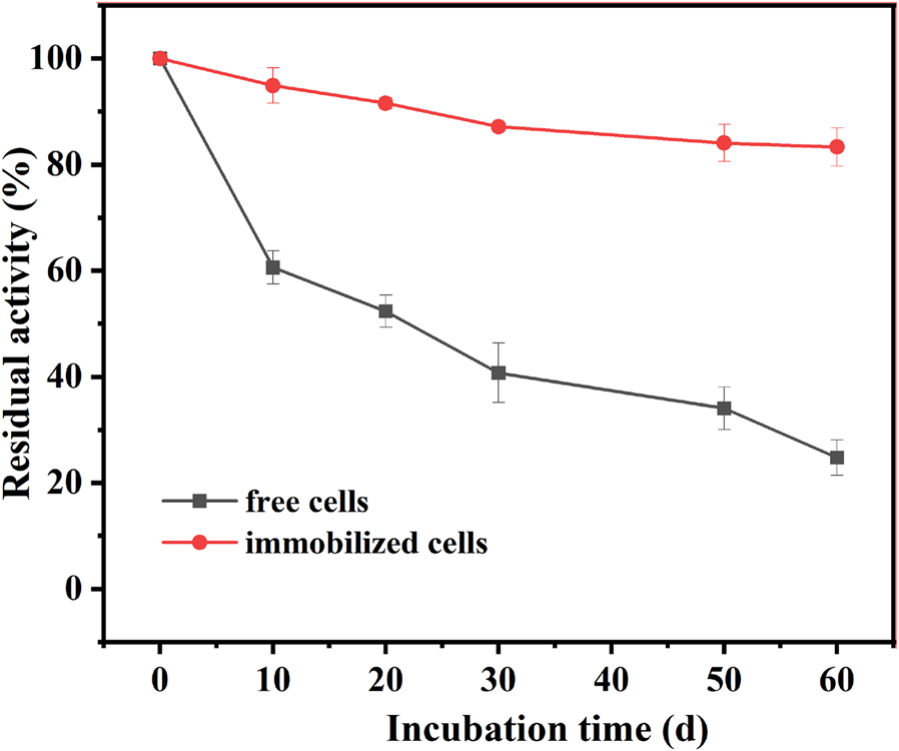
Storage stability of free and alginate-immobilized recombinant *E. coli* cells at 4 ℃. Reaction conditions: 4 mL Tris–HCl buffer (200 mM, pH 7.0), 100 mM MAA, 150 mM glucose, 30 mg/mL of free recombinant *E. coli* cells (wet weight), or immobilized cells containing the same amount of free cells, 40 ℃, 200 rpm

Since the storage stability of immobilized cells was of great significance to industrial reagent production, the catalytic ability at different periods of recombinant *E. coli* cells storing at 4 ℃ was monitored (shown in Fig. 5).

Free or immobilized recombinant *E. coli* cells were stored in Tris–HCl buffer at 4 ℃. The immobilized cells maintained more than 80% relative activity after 60 d, while the free cells remained only 24.7%. This showed that the storage stability of immobilized cells was better than that of free cells at 4 °C. Moreover, the product *e*.*e*. was maintained over 99.9%.

### Effect of substrate concentrations

**Fig. 6:**
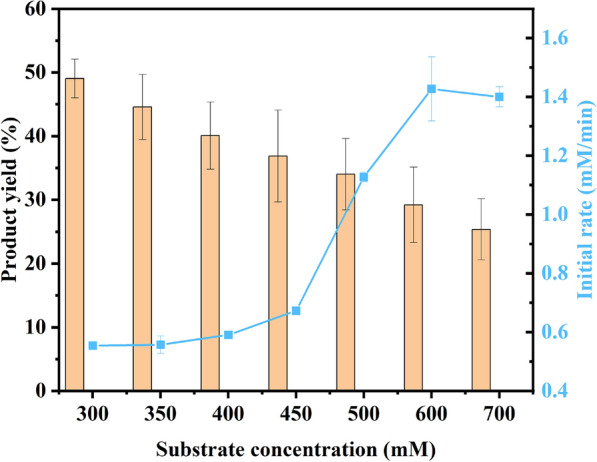
Effects of substrate concentrations on the bio-reduction of MAA to (*R*)-HBME with alginate-immobilized recombinant *E. coli* cells. Reaction conditions: 4 mL Tris–HCl buffer (200 mM, pH 7.0), 800 mM glucose, different concentrations of MAA (300, 350, 400, 450, 500, 600, 700 mM), 30 mg/mL free recombination *E. coli* cells (wet weight), or immobilized cells containing the same amount of free cells, 40 ℃, 200 rpm; (*n* = 3, different lowercase letters represent significant differences between groups, *p* < 0.05)

The success of industrial production mainly depended on the output of bioproducts as much as possible. However, substrate inhibition is a common phenomenon in the biocatalytic asymmetric reduction because there were different characteristics between substrates and cells. Some studies had proved that the substrate tolerance of immobilized cells was better than that of free cells (Wei et al. 2015). Therefore, the effects of initial substrate concentrations on the catalytic activity of immobilized cells were studied (shown in Fig. 6).

As shown in Fig. 6, when the substrate concentration varied from 300 mM (yield only of 55.4%) to 600 mM, the initial reaction rate was remarkably accelerated, but the maximum yield appeared to gradually decrease. The high substrate concentration greatly accelerated the rate of product formation. On one hand, it was speculated that the substrate concentration (600 mM) and the amount of catalyst at this point reached to saturation. When the substrate concentration increased, the initial reaction rate still remained basically unchanged. On the other hand, because cells are embedded in the carrier, the contact area with substrate is limited, resulting in catalytic saturation of substrate concentration. At the same time, the partial accumulation of gluconic acid caused the partial decrease of pH in the space of immobilized enzyme. So, the substrate concentration continued to increase, and yet the initial reaction rate did not increase. Whereas, the yield decreased with the increase of substrate concentration. This possibly results from the local concentration of gluconic acid, which has certain toxic and side effects on cells. As a result, the catalytic capacity of immobilized cells is reduced, so that the reaction rate cannot continue to increase. The reaction rate cannot continue to increase. Furthermore, the increment of investigated substrate concentrations did not alter the product *e*.*e*. (> 99.9%).

## Conclusions

In summary, a high-efficient immobilization recombinant *E.coli* cells system was developed for the asymmetric reduction of MAA to (*R*)-HBME. Compared with free cells, Ca-alginate cell beads led to increment of mass transfer resistance of substrate absorption and product release, reducing in initial reaction rate. However, immobilized cells had outstanding thermal stability, pH stability, storage stability, reusability and high substrate concentration tolerance. Besides, immobilized material was no negative effect on enantioselectivity of (*R*)-HBME (> 99.9%). Therefore, immobilized cells were more available for large-scale industrial applications.

## Data Availability

The data sets used in this study are available from the corresponding author on reasonable request.

## Abbreviations

(*R*)-HBME: Methyl-(*R*)-3-Hydroxybutyrate
MAA: Methyl Acetoacetate
*e*.*e*.: Enantiomeric excess
IPTG: Isopropyl-*β*-d-thiogalactoside
CS: Chitosan
CA: Calcium alginate
